# Biochemical Parameters as Prognostic Markers in Severely Ill COVID-19 Patients

**DOI:** 10.7759/cureus.28594

**Published:** 2022-08-30

**Authors:** Anjali Pitamberwale, Tariq Mahmood, Azmat Kamal Ansari, Shabana Andleeb Ansari, Kirti Limgaokar, Lalit Singh, Geeta Karki

**Affiliations:** 1 Biochemistry, Fergusson College, Pune, Maharashtra, IND; 2 Biochemistry, Teerthankar Mahaveer Medical College and Research Centre, Moradabad, Uttar Pradesh, IND; 3 Biochemistry, Shri Ram Murti Smarak Institute of Medical Sciences, Bareilly, Uttar Pradesh, IND; 4 Pathology, Shri Ram Murti Smarak Institute of Medical Sciences, Bareilly, Uttar Pradesh, IND; 5 Respiratory Medicine and Critical Care, Shri Ram Murti Smarak Institute of Medical Sciences, Bareilly, Uttar Pradesh, IND; 6 Anaesthesia and Critical Care, Shri Ram Murti Smarak Institute of Medical Sciences, Bareilly, Uttar Pradesh, IND

**Keywords:** multiorgan dysfunction, critically ill covid-19 patients, prognostication, renal dysfunction, inflammatory parameters, biochemical alterations, covid‐19

## Abstract

Background

Prognostication plays a pivotal role in critical care medicine. Its importance is indisputable in the management of coronavirus disease 2019 (COVID-19), as the presentation of this disease may vary from docile, self-limiting symptoms to lethal conditions. Amid the COVID-19 pandemic, much emphasis was initially placed on molecular and serological testing. However, it was realized later that routine laboratory tests also provide key information in terms of the severity of the disease and thus could be used to predict the outcome of these patients.

Methodology

The aim of our study was to evaluate the biochemical parameters as prognostic markers in severely ill COVID-19 patients. We carried out a retrospective, case-control study. The study population was comprised of all severely ill COVID-19 patients admitted between October 2020 and January 2021 at our level 3 COVID hospital. Cases were defined as the patients who expired despite treatment and all resuscitative measures as per the standard operating procedures (SOPs) of our COVID intensive care unit (ICU) while controls were defined as the patients that were transferred out of the COVID ICU for further recovery. The detailed history, findings of physical examination, vitals recorded by point of care testing (POCT) devices at our ICU, clinical diagnosis, and the results of the biochemical analysis were recorded in a specially designed pro forma. The biochemical parameters recorded at the time of admission were compared between the groups of controls and cases in order to evaluate their role as predictors of mortality using appropriate statistical methods. P-values less than 0.05 were considered statistically significant. For all the parameters that showed a statistically significant difference, receiver operating characteristics (ROC) analysis was done to assess the utility of biochemical parameters as predictors of mortality or survival. Areas under the curve (AUCs) of 0.6 to 0.7, 0.7 to 0.8, 0.8 to 0.9, and >0.9 were considered acceptable, fair, good, and excellent for discrimination, respectively.

Results

Of the 178 severely ill COVID-19 patients enrolled in the study, 86 were controls and 92 were cases (52% mortality). Serum urea (p<0.0001), creatinine (p=0.0019), aspartate transaminase (AST) (p=0.0104), lactate dehydrogenase (LDH) (p=0.0001), procalcitonin (PCT) (p=0.0344), and interleukin 6 (IL-6) (p=0.0311) levels were significantly higher (p<0.05), while total protein (p=0.0086), albumin (p<0.0001), and indirect bilirubin (p=0.0147) levels were significantly lower (p<0.05) in cases as compared to controls. The difference was statistically insignificant (p>0.05) for serum sodium, potassium, total and direct bilirubin, globulin, alanine transaminase (ALT), alkaline phosphatase (ALP), D-dimer, and ferritin. On ROC analysis, urea was fair (AUC=0.721), creatinine (AUC=0.698) and IL-6 (AUC=0.698) were acceptable predictors of mortality, while albumin (AUC=0.698) was an acceptable predictor of survival in severely ill COVID-19 patients during their intensive care stay.

Conclusion

Understanding the pathophysiological changes associated with the severity of COVID-19 in terms of an alteration of biochemical parameters is a pressing priority. Our study highlights the importance of routine laboratory tests in predicting outcomes in severely ill COVID-19 patients.

## Introduction

Prognostication plays a crucial role in the management of coronavirus disease (COVID-19) [1-8]. It not only allows the identification of individuals at risk of poor outcomes but also helps in planning timely interventions. With early prognosis and timely intervention, even severely ill COVID-19 patients can be saved [2,4,6,8,9]. There are various models to support the prognostication of patients with COVID-19. Though these models are reported to have reasonable predictive performance, all are quite complex [2,6]. A feasible marker for the accurate identification of severely ill COVID-19 patients (who are at risk of further deterioration) is still coveted [4,7].

COVID-19 is an infectious disease caused by severe acute respiratory syndrome (SARS ) coronavirus 2 (CoV-2) [1]. It is known to have a wide spectrum of clinical manifestations; from mild respiratory symptoms to pneumonia and, in more severe cases, multiple organ failure [1,3,4]. Mortality in severely ill cases of COVID-19 is associated with respiratory insufficiency and/or multiple organ failure [3,4]. The mechanisms of progression and mortality in COVID-19 are proposed to be unbalanced immune responses [10,11]. This hypothesis has been supported by the demonstration of relevant molecular alterations in these patients [10-14]. Molecular alterations in severely ill COVID-19 patients suggest that these individuals are not only at greater risk of abnormal immune responses but also susceptible to complications like respiratory insufficiency and multiple organ failure [2,4-7,10-16]. Such abnormal immune responses result in an increased serum concentration of many pro-inflammatory mediators [10,11]. Several biomarkers have been found to be altered (predominantly due to the effect of pro-inflammatory mediators) in correlation with the severity of COVID-19. Though these biomarkers are promising prognostic markers, their analysis requires a state-of-the-art set-up [2,4,6,13].

Laboratory tests are particularly useful in validating a diagnosis, predicting disease severity, and monitoring disease progression in patients with infectious diseases like COVID-19 [15-18]. Timely diagnostic assessment and implementation of reliable tests are integral components of the comprehensive management of severely ill COVID-19 patients. Routine laboratory parameters are known to be affected by pathophysiological alterations induced by various diseases and thus are employed conventionally for the effective monitoring of patients in critical care units [15]. However, evidence on the role of biochemical parameters as readily available, cost-effective prognostic markers in severely ill COVID-19 patients is still evolving. Even routine biochemical parameters can provide critical information regarding the severity of diseases and can support the appropriate clinical management of COVID-19 patients [2,5-7,13-19]. Therefore, we planned this study of biochemical parameters in severely ill COVID-19 patients to evaluate their role as prognostic markers of poor outcomes.

## Materials and methods

An observational, retrospective, case-control study was conducted after obtaining approval from the Institutional Ethics Committee (IEC) of Shri Ram Murti Smarak Institute of Medical Sciences. We utilized data from all the patients admitted to the COVID intensive care unit (ICU) of our hospital, Shri Ram Murti Smarak (SRMS) Institute of Medical Sciences (IMS), Bareilly, Uttar Pradesh, India, from 1st October 2020 to 31st January 2021. As per the standard operating procedures (SOPs) of our institute, patients with clinical syndromes associated with COVID-19 infection (referred from the emergency department or wards of other departments) were admitted to our COVID ICU. These patients were screened (based on the following inclusion and exclusion criteria) for their eligibility as study participants [16]. Only severely ill COVID-19 patients admitted to our COVID ICU were included in our study.

The following diagnostic definitions were used to define severely ill COVID-19 patients:

1. COVID-19 Patients

Defined as patients with clinical syndromes associated with COVID-19, who tested positive for either real-time-polymerase chain reaction (RT-PCR) TrueNat or Rapid Antigen Test (RAT) for COVID-19 [1,6,17].

2. Severely Ill COVID-19 Patients

Our ICU follows the "Revised Guidelines on Clinical Management of COVID-19", issued by the Government of India Ministry of Health & Family Welfare Directorate General of Health Services (EMR Division) to define severely ill COVID-19 patients [3,16,18,19]. According to these guidelines, severely ill COVID-19 patients are defined as laboratory-confirmed cases of COVID-19 with at least one of the following:

(A) Severe pneumonia: Defined as a patient suspected of having respiratory tract infection, with one or more of the following: 1) Respiratory rate greater than 30 breaths per minute; 2) Severe respiratory distress; 3) Peripheral capillary oxygen saturation (SPO_2_) less than 90 percent (%) on room air [3,16,18,19].

(B) Acute respiratory distress syndrome (ARDS): Defined as follows: 1) The development of new or worsening respiratory symptoms within one week of known exposure; 2) Bilateral opacities not explained by chest imaging studies; 3) Respiratory failure caused by factors other than cardiac failure or fluid overload; 4) Mild, moderate or severe ARDS: Defined as (a) Partial pressure of oxygen in arterial blood (PaO_2_) less than 200 (millimeters of mercury) mmHg or fraction of inspired oxygen (FiO_2_) less than or equal to 300 mmHg (with positive end-respiratory pressure (PEEP) or continuous positive airway pressure (CPAP) more than or equal to 5 centimeters (cm) of water (H_2_O)); (b) When PaO2 is not available, SpO_2_ or FiO_2_ less than or equal to 315; (c) Respiratory distress in non-ventilated patients [3,16,18,19].

(C) Sepsis: Defined as a dysregulated host response to infection resulting in life-threatening organ dysfunction, diagnosed by: 1) Symptoms like altered mental status, difficult or fast breathing, or skin mottling; 2) Signs like low oxygen saturation, reduced urine output, fast heart rate, weak pulse, cold extremities or low blood pressure; 3) Laboratory evidence like coagulopathy, thrombocytopenia, acidosis, high lactate, or hyperbilirubinemia [3,16,18,19].

(D) Septic shock: Defined as persisting hypotension despite appropriate volume resuscitation, requiring vasopressors in order to maintain mean arterial pressure (MAP) more than or equal to 65 mmHg and serum lactate level less than 2 mmol/L [3,16,18,19].

We excluded the following patients from our study: 1) Suspected cases of COVID-19 with no confirmatory laboratory results; 2) Laboratory confirmed COVID-19 cases that do not meet our criteria for severely ill COVID-19 patients; 3) Patients with insufficient data for further analysis, who denied the standard treatment protocol of our hospital or discharged against medical advice.

Admission, history-taking, physical examination, analysis (done either by point of care testing (POCT) devices at the ICU or autoanalyzers at the laboratory), and management of all patients were done following the SOPs of our institute. Data required for the study were retrieved from patients’ clinical case files archived at the COVID ICU and our laboratory and hospital information systems (LIS and HIS), version 3. These endogenous information systems were developed by professionals of our engineering college (SRMS College of Engineering, Technology & Research, Bareilly) in 2017. Patients’ clinical profiles (detailed history, findings of physical examination, vitals from POCT devices, radiological findings, and diagnosis) and laboratory results of blood samples collected at the time of admission to the COVID ICU were recorded in an especially designed pro forma. All the biochemical parameters (bilirubin (total, direct, and indirect), aspartate transaminase (AST), alanine transaminase (ALT), alkaline phosphatase (ALP), lactate dehydrogenase (LDH), urea, creatinine, sodium (Na+), and potassium (K+)) were analyzed in the biochemistry section while D-dimer and procalcitonin (PCT) estimation was done at the pathology section of our Central Clinical Laboratory. Serum interleukin-6 (IL-6) and ferritin levels were measured in the Chemiluminescence Immunoassay (CLIA) section of our Central Research Laboratory. All parameters were analyzed following the SOPs of the respective sections of our laboratories. Table 1 summarizes the details of the platforms and the methods (along with their respective system packs) used for all laboratory parameters of our study. Results of all quantitative analyses were validated by means of internal and external quality control procedures according to the SOPs of respective sections of our laboratories.

**Table 1:** Instruments and Methods Used for the Quantitative Analysis of Biochemical Parameters

Sr.No.	Instruments	Tests	Reference Intervals	Methods
A.	Mindray BS-380: Fully-Automated Analyzer (Serum in plain vacutainer)	Liver Function Tests		
		Total Bilirubin	0.2-1.2 Milli Grams (mg) per Deciliters (dl)	Diazotized Sulfanilic Acid (DSA)
		Direct Bilirubin	0-0.3 mg/dl	DSA
		Indirect Bilirubin	0.5-0.9 mg/dl	Calculated
		Serum Protein	6.6-8.3 Grams (g) per dl	Biuret
		Serum Albumin	3.5-5.0 g/dl	Bromo Cresol Green (BCG)
		Serum Globulin	2.5-3.5 g/dl	Calculated
		Alanine Aminotransferase (ALT)	10-46 International Units (IU) per Liter (L)	Creatine Kinase (CK) activity assay (International Federation of Clinical Chemistry (IFCC)/Kinetic)
		Aspartate Aminotransferase (AST)	10-49 IU/L	CK activity assay (IFCC/Kinetic)
		Alkaline Phosphatase (ALP)	108-306 IU/L	Para Nitrophenyl Phosphate (PNP) and AMP Buffer (IFCC/Kinetic)
		Lactate Dehydrogenase (LDH)	M: 248 IU/L F: 247 IU/L	IFCC/Kinetic
		Renal Function Tests		
		Urea	20-40 mg/dl	Urease-Glutamate dehydrogenase (GLDH)
		Creatinine	M: 0.6-1.4 mg/dl F: 0.6-1.2 mg/dl	Sarcosine Oxidase
B.	Avantor Easylyte: Electrolyte Analyzer (Serum in plain vacutainer)	Sodium	135.0-145.0 Milli Moles (mmol) per L	Ion-selective electrodes (ISE)
		Potassium	3.5-5.0 mmol/L	Ion-selective electrodes (ISE)
C.	Beckman Coulter Access 2: Immunoassay System (Serum in plain vacutainer)	Interleukin (IL)-6	<6.4 Pico Grams (pg) per ml	Chemiluminescent Immunoassay method (CLIA)
		Ferritin	M: 30-350 Nano Grams (ng) per ml F: 20-250 ng/ml	CLIA
D.	AQT90 Flex: Immunoassay Analyzer (Whole blood in EDTA Vacutainer)	D-Dimer	Upto 50 Years: 80-583 µg/L Above 50 Years: 80-654 µg (Micro Grams)/L	Immunoassay (IA)
		Procalcitonin (PCT)	<0.15 ng/ml	IA

We categorized the data of the subjects based on their outcome (Survivors or Non-survivors) during their stay in the ICU. The survivors were designated as the control group while the non-survivors were the case group. We compared the results of the blood samples collected at the time of admission to the COVID ICU between the controls and cases to study the role of these biochemical markers in the prediction of mortality in severely ill COVID-19 patients.

Categorical variables were described as frequency and percentages using demographics and continuous variables as mean and standard deviation (SD). Quantitative data were assessed for linearity using Kolmogorov-Smirnov analysis and tests of statistical significance (student’s unpaired t-test or Mann-Whitney-U test) were used depending upon the data type. Means for continuous variables were compared using independent group p-values in MedCalc software. The parameters with p-values less than 0.05 were considered statistically significant. Receiver operating characteristics (ROC) curve analysis was utilized (for all the parameters that showed a statistically significant difference between the cases and controls) to assess the utility of biochemical parameters to predict mortality or survival. AUC of 0.6 to 0.7, 0.7 to 0.8, 0.8 to 0.9, and >0.9 were considered acceptable, fair, good, and excellent discrimination, respectively. Further, for all parameters with an AUC > 0.6, the sensitivity and specificity as predictors of survival or mortality (at specific cut-offs) were also assessed on the basis of the ROC analysis.

## Results

**Figure 1:**
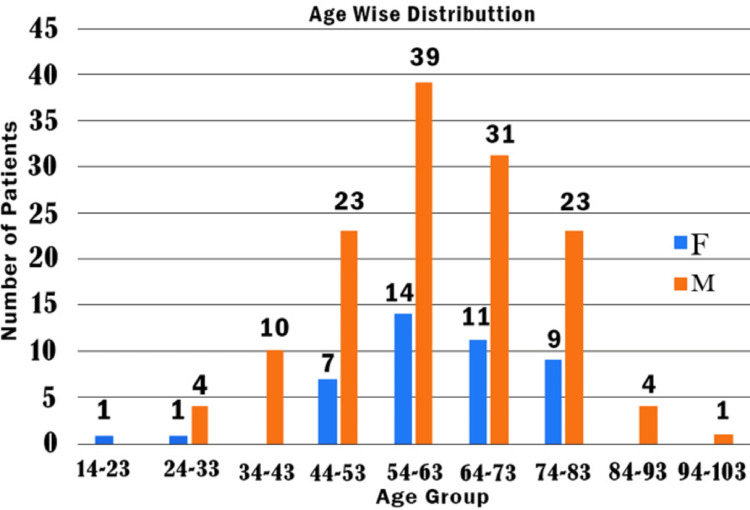
Age-Wise Distribution of Study Participants

Out of 231 critically ill patients admitted to our COVID ICU between October and January 2021, 178 severely ill COVID-19 patients were enrolled in the present study (based on our inclusion). We excluded 53 patients (2 cases without a confirmatory laboratory result for confirmation of the diagnosis of COVID 19, 16 cases which did not meet our COVID ICU criteria to be classified as severely ill COVID-19 patients, 7 patients who did not have sufficient data for further analysis and 28 patients that took discharge against medical advice) based on our exclusion criteria. Out of 178 patients enrolled in the study, 134 (75.28%) were males and 44 (24.72%) were females. The youngest patient was 14 years old and the oldest patient was 97 years old, while the average age was found to be 62 years. The greatest number of patients (74.72%) belonged to the age group of 51-80 years. Figure 1 and Table 2 summarise the age-wise distribution of patients in our study.

**Table 2:** Mean Age, Gender Distribution, and Outcome (in Terms of Survival or Mortality)

Total Subjects - 178		
Age Group Range (14-97 years)		
Males (n=134)	Mean=61 years	
Females (n=44)	Mean=62 years	
Outcome		
	Survived (n=86)	Expired (n=92)
Males (n=134)	63 (47.02%)	71 (52.98%)
Females (n=44)	23 (52.27%)	21 (47.73%)

We categorized 178 patients enrolled in our study based on their outcomes (survivors or Non-survivors) during their stay in the ICU. Table 2 summarizes the gender distribution and outcomes of the study population. In our study, 86 (48.31%) were survivors and 92 (51.68%) were non-survivors. The survivors were labeled as controls while non-survivors were labeled as cases. Mortality was found to be around 52% among the study population. The average age of controls was 59 years, while among cases, it was 63 years. Out of 134 males, 63 (47.02%) and 71 (52.98%) were controls and cases, respectively. Out of 44 females, 23 (52.27%) and 21 (47.73%) were controls and cases, respectively.

The biochemical parameters showed statistically significant differences (p<0.05) in their average values between the cases and controls groups. A statistically significant difference was observed for serum urea (p<0.0001), creatinine (p=0.0019), indirect bilirubin (p=0.0147), serum protein (p=0.0086), albumin (p<0.0001), AST (p=0.0104), LDH (p=0.0001), PCT (p=0.0344), and IL-6 (p=0.0311) (Table 3). Serum urea, creatinine, AST, LDH, PCT, and IL-6 were significantly higher (p<0.05), while total protein, albumin, and indirect bilirubin were significantly lower (p<0.05) in cases as compared to controls. The difference was statistically insignificant (p>0.05) for serum sodium, potassium, total and direct bilirubin, globulin, ALT, ALP, D-dimer, and ferritin.

**Table 3:** Comparison of Biochemical Parameters Between the Groups of Controls and Cases * p-value less than 0.05 (p<0.05)

S.No.	Parameter	Average values of parameters		p-value
		Group I: Survivors (Controls)	Group II: Non-survivors (Cases)	
	Urea (mg/dl)	64.65	93.30	p < 0.0001*
	Creatinine (mg/dl)	1.41	1.98	p = 0.0019*
	Na^+^(mmol/l)	135.22	135.49	p = 0.5153
	K^+^ (mmol/l)	4.32	4.42	p = 0.7850
	Total Bilirubin (mg/dl)	1.33	1.06	p = 0.2676
	Direct Bilirubin (mg/dl)	0.97	0.61	p = 0.5936
	Indirect Bilirubin (mg/dl)	0.36	0.46	p = 0.0147*
	Serum Protein (g/dl)	6.30	6.14	p = 0.0086*
	Albumin (g/dl)	3.56	3.14	p < 0.0001*
	Globulin (g/dl)	4.33	3.31	p = 0.8474
	AST (IU/L)	69.30	89.69	p = 0.0104*
	ALT (IU/L)	53.91	59.34	p = 0.6850
	ALP (IU/L)	109.87	136.36	p = 0.3075
	LDH (IU/L)	467.55	601.84	p = 0.0001*
	PCT (ng/ml)	0.82	4.19	p = 0.0344*
	IL-6 (pg/ml)	68.54	931.00	p = 0.0311*
	D-Dimer (µg/ml)	6675.22	7234.83	p = 0.6089
	Ferritin (ng/ml)	740.14	913.34	p = 0.2532

Receiver operating characteristics (ROC) curves were plotted for all the parameters that showed a statistically significant difference between the cases and controls. Below, we are reporting only the results of the ROC curve analysis for the parameters that were found to be predictors of mortality or survival based on AUC (Table 3).

**Figure 2:**
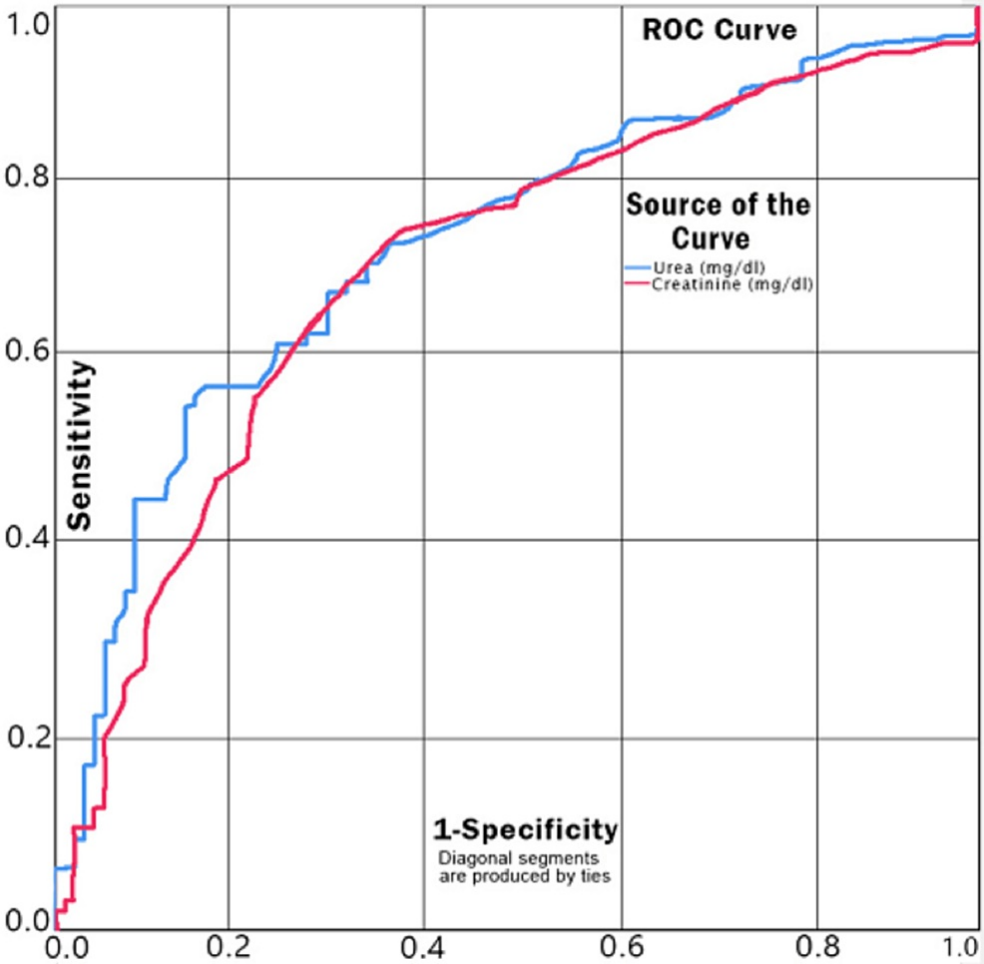
ROC Curve for Serum Urea and Creatinine as Predictors of Mortality ROC: receiver operating characteristic

The ROC curve for serum urea and serum creatinine as predictors of mortality has an area under the curve (AUC) of 0.721 (fair predictor) and 0.698 (acceptable), respectively. For a cut-off value of 52 mg/dl for serum urea, the sensitivity and specificity are 0.736 and 0.605, respectively, while for a cut-off value of 0.95 mg/dl for serum creatinine level, the sensitivity and specificity are 0.747 and 0.605, respectively, as a predictor of mortality (Figure 2).

**Figure 3:**
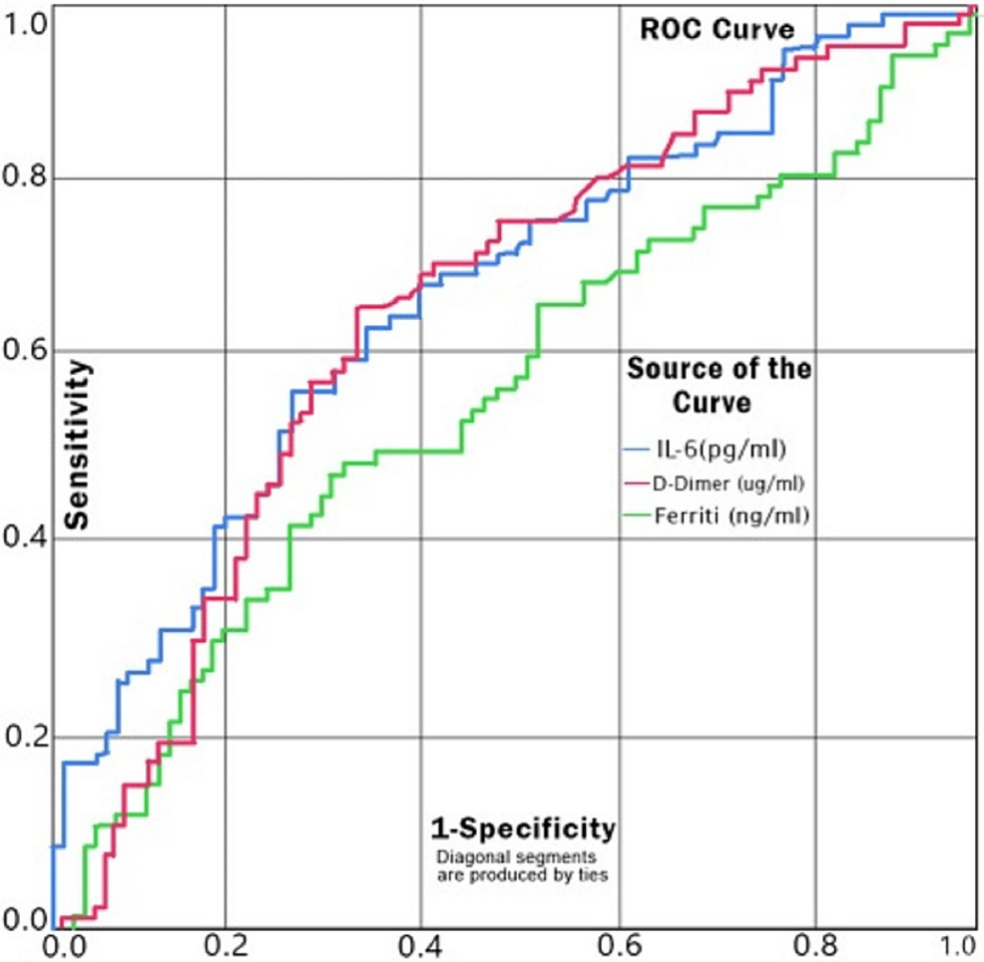
ROC Curve for Serum IL-6 as a Predictor of Mortality ROC: receiver operating characteristic

The ROC curve for serum IL-6 as a predictor of mortality has an AUC of 0.698 (p=0.0311), which puts it at the upper bounds of an acceptable score. For a cut-off value of 44.41 pg/ml of serum IL-6, the sensitivity and specificity are 0.703 and 0.605, respectively, as a predictor of mortality (Figure 3).

**Figure 4:**
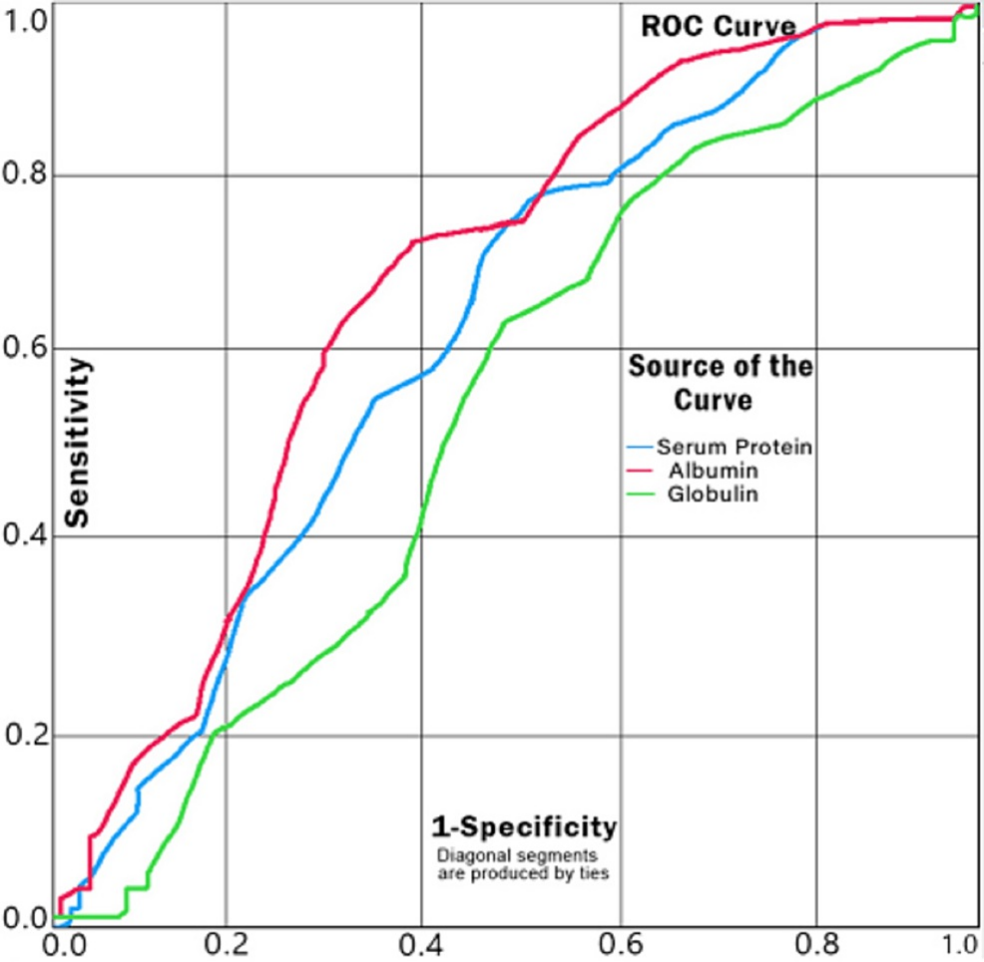
ROC Curve for Serum Albumin as a Predictor of Survival ROC: receiver operating characteristic

The ROC curve for serum albumin as a predictor for survival has an AUC of 0.698 (p< 0.0001), which puts it at the upper bounds of an acceptable score. For a cut-off value of 3.25 g/dl of serum albumin, the sensitivity and specificity are 0.767 and 0.593, respectively, as predictors of survival (Figure 4).

## Discussion

In our study, critically ill COVID-19 patients showed distinct clinical, demographic, and laboratory features at the time of ICU admission. It was observed that men are more affected by COVID-19 in terms of both morbidity and mortality. Previous studies showed that this could be due to the expression of more angiotensin-converting enzyme 2 (ACE2) receptors in men than in women [20]. Moreover, females are reported to have more resistance to fight infections than males, which is possibly mediated by differences in sex hormones as well as immune systems [20,21]. In our study, the representation of women among severely ill COVID-19 patients was much higher in the menopausal age groups. This can be explained by the effect of estrogen on the expression of ACE2 receptors. Estrogen is known to exert a protective effect against cardiovascular diseases in women in the reproductive age group by means of its modulatory effects on the expression of ACE2 receptors. This phenomenon may also be relevant for their protection against COVID-19, especially in the reproductive age group [20,21]. The average age of the population in our study was 62 years. The largest number of patients (26.40%) belonged to the 51-60 years of age group. The average age of those who survived was 59 years while the average age of those who succumbed to the illness was 63 years. Earlier studies have also suggested an increased risk of COVID-19-related mortality among individuals 60 years or older [21,22].

Serum urea (p<0.0001),creatinine (p=0.0019), AST (p=0.0104), LDH (p=0.0001), procalcitonin (PCT) (p=0.0344), and IL-6 (p=0.0311) levels were significantly higher (p<0.05) while total protein (p=0.0086), albumin (p<0.0001), and indirect bilirubin (p=0.0147) levels were significantly lower (p<0.05) in cases as compared to controls.The difference was statistically insignificant (p>0.05) for serum sodium, potassium, total and direct bilirubin, globulin, ALT, ALP, D-dimer, and ferritin.

Abnormal renal function tests (higher levels of serum urea and creatinine) were reported in our study and the difference was found to be highly significant (urea (p<0.0001) and creatinine (p=0.0019)) between the controls and cases. The ROC curve for urea (AUC=0.721) and creatinine (AUC=0.698) were found to be fair and acceptable predictors of mortality, respectively. For a cut-off value of 52 mg/dl for urea level, the sensitivity and specificity are 0.736 and 0.605, respectively, while for a cut-off value of 0.95 mg/dl for creatinine level, the sensitivity and specificity were 0.747 and 0.605, respectively (Figure 2). In several studies, renal dysfunction has been correlated with mortality in COVID-19 patients [23,24]. The SARS-CoV-2 infection itself has been suspected to cause renal dysfunction. However, it may also cause worsening of pre-existing chronic renal disease [20]. All these facts suggest renal dysfunction-induced mortality in COVID-19 patients.

Earlier studies have shown that increased levels of IL-6 are related to increased disease severity and resultant mortality in COVID-19 [25]. As reported by many, higher levels of inflammatory parameters like IL-6 can predict poor outcomes; our study also supports this finding. Elevation in levels of serum IL-6 in the cases (p=0.0311) was reported in our study. The ROC curve of IL-6 shows AUC=0.690 suggesting it to be an acceptable predictor of mortality. For the cut-off value of 44.41 pg/ml of IL-6 level, the sensitivity and specificity were found to be 0.703 and 0.605, respectively.

Higher albumin levels are associated with greater chances of survival as reported by some studies [24]. Studies have also shown that serum albumin concentrations tend to decrease with age in both males and females [22]. Albumin has also been shown to have a modulatory effect on ACE2 expression and to down-regulate insulin-mediated ACE2 expression in cultured podocytes [20,21]. This could possibly be a mechanistic pathway for the increased severity of the disease with falling serum albumin levels. Our findings have shown that higher albumin levels (p<0.0001) are a predictor of survival. The ROC curve shows that it was an acceptable (AUC=0.698) predictor of survival. At the cut-off value of 3.25 g/dl of albumin level, the sensitivity and specificity are 0.767 and 0.593, respectively, as predictors of survival.

The difference between controls and cases for other biochemical parameters (sodium, potassium, total and direct bilirubin, globulin, ALT, ALP, D-dimer, and ferritin) was found to be statistically insignificant (p-value>0.05). Many of these (especially D-dimer) are reported to have good prognostic value in several studies [26].

Based on the findings of our study, we propose biochemical parameters as practical prognostic markers in severely ill COVID-19 patients due to the routine practice of their monitoring (owing to their easy availability) in these patients.

We assume that the manifestation of clinical syndromes associated with severe COVID-19 is the actual time when the development of metabolic alterations responsible for death in severely ill COVID-19 patients is triggered. Based on this, we postulate that prognosticating at this triggering moment has to be both sensitive and specific [4,7,12,16,18,19]. Theoretically, by this time, the biochemical alterations related to poor prognosis might have become significantly distinct. Thus, we can detect them by virtue of suitable prognostic markers. Sampling earlier will adversely affect the accuracy and precision of prognostication, as the mortality-determining pathophysiological processes may yet reach a detectable threshold. Prognosticating after the development of full-fledged multiorgan failure will be futile as the chance for appropriate interventions in order to prevent mortality and/or morbidity would have been wasted [8]. However, it has to be noted that since COVID-19 is a relatively new disease, the data regarding the underlying biochemical alterations as a result of pathophysiological events responsible for multiorgan damage and thus poor outcomes is yet to be clearly unraveled. Further research into the pathophysiological processes responsible for poor outcomes and biochemical alterations associated with these processes, especially in the context of the development of clinical syndromes associated with severe illness, multiorgan failure, and mortality in COVID-19 patients, is absolutely necessary.

Based on the observations of this study, we believe that routine biochemistry parameters can be used as feasible prognostic markers in severely ill COVID-19 patients. With the help of easily available prognostic markers, COVID-19 patients at higher risk of poor outcomes can be appropriately managed [6-9]. With timely prognostication and interventions, the utilization of available resources can be improved [9]. Judicious utilization of available resources is very important, especially in pandemics like COVID-19.

The main strength of this study is its planning and execution at an ICU known for its quality services and adherence to SOPs and protocols. The well-planned study (appropriate inclusion and exclusion criteria, adequate sample size, and statistical analysis by appropriate methods), the inclusion of results of the investigation done at the time of identification of clinical syndromes associated with severe COVID-19 in all study participants, and the quantitative analysis of all biochemical parameters using cutting-edge methods are some of the positive aspects of our study [2,4,8,12,16,18,19,27]. More importantly is the fact that the study was focused on the utility of biochemical parameters that are simple, feasible, cost-effective, and easily available at most critical care units where these patients are primarily managed.

The main limitation of this study is its retrospective design. However, we assume that the accuracy of the findings is acceptable, as we followed the SOPs prevalent in our institute for both biochemical investigations as well as the management of all patients enrolled in the study. The limited sample size and inclusion of patients with pre-existing diseases (including those who required invasive ventilation) are the major limitations. Studies with a larger sample size, with a special emphasis on understanding the role of other factors (like pre-existing diseases and the type of ventilatory support required during intensive care, etc.) on the biochemical profile of these patients and their correlation with adverse outcomes are direly needed. A study of these biochemical parameters in mild and moderate cases of COVID-19 as well would provide a better understanding of these biochemical alterations and their relation to the development of severe illness. Understanding these biochemical alterations would not only help in prognosticating the risk of development of severe illness but also guide the development of better treatment protocols [2,7,19,28,29]. Clearance of biochemical parameters has emerged as an important prognostic marker in several critical illnesses [28-29]. Serial measurements of these biochemical parameters in patients with COVID-19 will reflect their clearance and thus aid in deciding the most appropriate timing of their analysis for better prognostication. Further research on derived scores from a combination of the promising prognostic biochemical parameters (serum urea, creatinine, bilirubin, protein, albumin, AST, LDH, PCT, and IL-6) of our study might provide additional prognostic clues.

## Conclusions

Renal dysfunction (elevated levels of serum urea and creatinine) and elevated serum IL-6 levels but lower serum albumin levels were found to be the best prognostic markers of in-hospital mortality of severely ill COVID-19 patients in our study population. We propose that the vigilant monitoring of these biochemical parameters can aid in the prognostication of COVID-19 and thus improve the clinical management of these high-risk patients. However, large‐scale, multicenter studies designed to evaluate the prognostic utility of these parameters in the prognostication of COVID-19, especially in severely ill patients, are required to validate our findings. It would really be very useful if these routine, time-tested, and feasible biochemical parameters were validated to be prognostic markers in severely ill COVID-19 patients.
